# BRAT1 links Integrator and defective RNA processing with neurodegeneration

**DOI:** 10.1038/s41467-022-32763-6

**Published:** 2022-08-26

**Authors:** Zuzana Cihlarova, Jan Kubovciak, Margarita Sobol, Katerina Krejcikova, Jana Sachova, Michal Kolar, David Stanek, Cyril Barinka, Grace Yoon, Keith W. Caldecott, Hana Hanzlikova

**Affiliations:** 1 Laboratory of Genome Dynamics, Institute of Molecular Genetics of the Czech Academy of Sciences, 142 20 Prague 4, Czech Republic; 2 Faculty of Science, Charles University in Prague, 128 43 Prague 2, Czech Republic; 3 Laboratory of Genomics and Bioinformatics, Institute of Molecular Genetics of the Czech Academy of Sciences, 142 20 Prague 4, Czech Republic; 4 Laboratory of RNA Biology, Institute of Molecular Genetics of the Czech Academy of Sciences, 142 20 Prague 4, Czech Republic; 5 Laboratory of Structural biology, Institute of Biotechnology of the Czech Academy of Sciences, BIOCEV, 252 50 Vestec, Czech Republic; 6 Department of Pediatrics, Division of Clinical and Metabolic Genetics, The Hospital for Sick Children, University of Toronto, Toronto, ON M5G 1X8 Canada; 7 Genome Damage and Stability Centre, School of Life Sciences, University of Sussex, Falmer, Brighton, BN1 9RQ UK

**Keywords:** Neuroscience, Cell biology

## Abstract

Mutations in *BRAT1*, encoding BRCA1-associated ATM activator 1, have been associated with neurodevelopmental and neurodegenerative disorders characterized by heterogeneous phenotypes with varying levels of clinical severity. However, the underlying molecular mechanisms of disease pathology remain poorly understood. Here, we show that BRAT1 tightly interacts with INTS9/INTS11 subunits of the Integrator complex that processes 3’ ends of various noncoding RNAs and pre-mRNAs. We find that Integrator functions are disrupted by BRAT1 deletion. In particular, defects in BRAT1 impede proper 3’ end processing of UsnRNAs and snoRNAs, replication-dependent histone pre-mRNA processing, and alter the expression of protein-coding genes. Importantly, impairments in Integrator function are also evident in patient-derived cells from *BRAT1* related neurological disease. Collectively, our data suggest that defects in BRAT1 interfere with proper Integrator functions, leading to incorrect expression of RNAs and proteins, resulting in neurodegeneration.

## Abstract (web summary)

Mutations in BRAT1 are associated with neurodevelopmental delay and neurodegeneration. Here, the authors show that BRAT1 is a component of Integrator and is important for processing of specific RNAs. They further demonstrate that BRAT1 mutant patient-derived cells exhibit reduced levels of the Integrator catalytic subunit and increased levels of misprocessed UsnRNAs and impaired RNA processing.

## Introduction

Mutations in *BRAT1*, encoding BRCA1-associated ATM activator 1, a protein implicated in the cellular response to DNA damage and maintenance of mitochondrial homeostasis^1–3^, were initially associated with lethal neonatal rigidity and multifocal seizure syndrome (RMFSL, OMIM 614498), a neurological disorder characterized by microcephaly, hypertonia, epilepsy, seizures and death within two years of birth^4–6^. Subsequently, *BRAT1* mutations were also identified in patients with milder clinical forms including neurodevelopmental disorder with cerebellar atrophy and with or without seizures (NEDCAS, OMIM 618056), epilepsy of infancy with migrating focal seizures (EIMFS), and congenital ataxia (CA)^7–10^. Recently, we and others associated mutation of *BRAT1* with nonprogressive cerebellar ataxia (NPCA), which is among the mildest form of BRAT1-associated disease identified to date^11,12^. However, the molecular role of BRAT1 and the mechanism/s by which mutations in this gene trigger a variety of neurological disorders remain unknown.

Similar to BRAT1, mutations in the human Integrator complex are associated with a severe recessive human neurodevelopmental syndrome, which is characterized by profound intellectual disability, epilepsy and subtle structural brain abnormalities^13,14^. Integrator is a multi-subunit protein complex that interacts with and is functionally associated with RNA polymerase II (RNAPII)^15,16^. Integrator is conserved across metazoans and is composed of at least 14 subunits, denoted INTS1 to INTS14, with a combined molecular weight of more than 1.5 MDa^17,18^. Most Integrator complex subunits lack obvious homology with any RNA processing proteins or transcriptional regulators that would allow predicting their molecular function, with the exception of INTS9 and INTS11. INTS9 and INTS11 display sequence homology with the CPSF-100 and CPSF-73 subunits of the cleavage and polyadenylation specificity factor, respectively, which form the endonuclease factor responsible for cleavage of pre-mRNA at the polyA site^19–22^. INTS11, like CPSF-73, has a β-lactamase/β-CASP domain and harbors RNA endonuclease activity. INTS11 forms a heterodimer with INTS9, a catalytically inactive homolog, that binds a scaffold protein INTS4 to form the core catalytic cleavage module of Integrator^23^. This complex can cleave various nascent noncoding non-polyadenylated RNA, such as small nuclear RNAs (UsnRNAs)^15^, enhancer RNAs (eRNAs)^24^, long-noncoding RNAs (lncRNAs)^25,26^, and a human telomerase-associated RNA^27^, as part of their processing to their mature form in a mechanism that is intimately coupled with transcription termination. Moreover, Integrator modulates the expression of specific protein-coding genes by cleaving nascent RNAPII transcripts and regulating transcription elongation through pause/release cycle of RNAPII, especially at the promoter-proximal pause site^28–32^.

Here, we demonstrate that BRAT1 interacts with and stabilizes the Integrator cleavage heterodimer INTS9/INTS11, and is important for an efficient Integrator function. Moreover, we show that patient cells from BRAT1 related cerebellar ataxia exhibit increased levels of unprocessed UsnRNAs and alter gene expression and RNA processing. Collectively, these data identify BRAT1 as a factor important for the efficient functioning of the Integrator, thus linking the neuropathology of patients with the BRAT1 mutation and patients with the Integrator mutations.

## Results

### BRAT1 interacts with the Integrator catalytic cleavage heterodimer

**Fig. 1:**
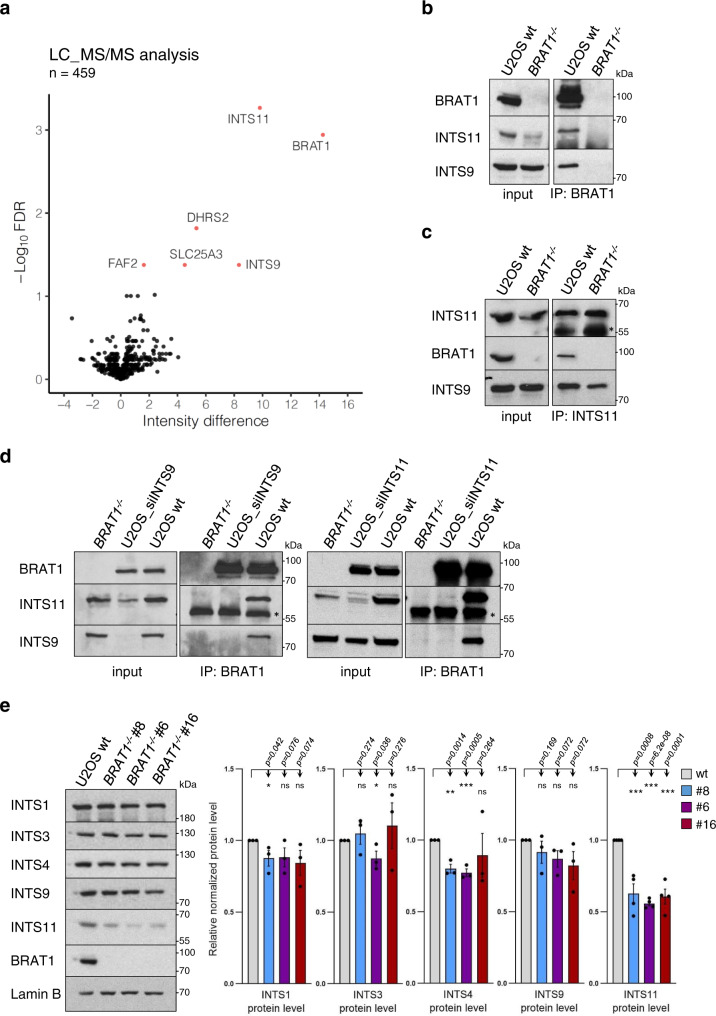
BRAT1 interacts with the Integrator catalytic cleavage heterodimer. **a** A volcano plot of proteins enriched in BRAT1 immunoprecipitates from U2OS cells. The –Log_10_ (Student’s *t*-test FDR) is plotted against the difference of mean intensities between wild-type and *BRAT1*^*−/−*^ samples. *Red dots* indicate significantly enriched proteins (intensity difference >1; FDR < 0.05). **b**, **c** Levels of BRAT1, INTS11 and INTS9 in BRAT1 (**b**) and INTS11 (**c**) immunoprecipitates from wild-type (U2OS wt) and *BRAT1*^*-/-*^ (clone #8) cells, measured by Western blotting. The *black asterisk* marks a nonspecific band. **d** Levels of BRAT1, INTS11 and INTS9 in BRAT1 immunoprecipitates from *BRAT1*^*−/−*^ (clone #8), U2OS wt, and U2OS cells transiently transfected with siINTS9 or siINTS11 as indicated. The *black asterisk* marks a nonspecific band. **b**–**d** The experiment was performed three times with similar results. **e** Immunoblot of indicated proteins and quantification in U2OS wt and *BRAT1*^*−/−*^ cells (clones #8, #6, and #16). Represented as the mean ± SD (*n* = 3 for INTS1, INTS3, INTS4 and INTS9; *n* = 4 for INTS11). Statistical significance was determined by a one-sided paired Student’s *t*-test (*p < 0.05, **p < 0.01, ***p < 0.001, ns not significant). Samples derived from the same experiment and blots were processed in parallel. Uncropped and unprocessed scans are provided in the Source Data file.

Recently, we identified a missense homozygous mutation in BRAT1 (p.V62E) that is associated with cerebellar atrophy, ataxia, ocular motor apraxia, and mild cognitive impairment, somewhat similar to that observed in DNA repair-defective diseases^11^. Since we did not detect defects in the activity of the ATM protein kinase, a master regulator of DNA damage response, in the *BRAT1*-mutated patient cells we aimed to elucidate other possible BRAT1 functions that might explain this disease phenotype and attempted to identify BRAT1 interacting partners. We therefore immunoprecipitated endogenous BRAT1 from U2OS cells using a BRAT1-specific antibody and identified co-precipitating proteins by mass spectrometry (MS). As a control, we generated and employed U2OS cell lines in which BRAT1 was deleted using CRISPR/Cas9-mediated genome editing (denoted *BRAT1*^*−/−*^) (Supplementary Fig. 1), to distinguish genuine partners of BRAT1 from those that were bound to the antibody non-specifically. This approach resulted in identification of INTS9 and INTS11, two subunits of Integrator complex, as putative BRAT1 interactors (Supplementary Data 1). These were the most abundant proteins identified by MS and were selected for further analysis (Fig. 1a).

To validate the data obtained from MS we immunoprecipitated endogenous BRAT1 or INTS11 from wild-type and *BRAT1*^*−/−*^ U2OS cells and conducted western blotting. These experiments confirmed the presence of both INTS9 and INTS11 Integrator subunits in BRAT1 immunoprecipitates from wild-type U2OS cells, and importantly their absence from parallel immunoprecipitates from *BRAT1*^*−/−*^ cells (Fig. 1b). Similarly, BRAT1 was present in INTS11 immunoprecipitates from wild-type cells (Fig. 1c). In addition, siRNA-mediated depletion of either INTS9 or INTS11 prevented co-immunoprecipitation of both subunits with BRAT1, implying that BRAT1 interacts with the assembled INTS9/INTS11 catalytic heterodimer (Fig. 1d). Importantly, the interaction of BRAT1 with INTS11 was detected in both nuclear and cytoplasmic extracts (Supplementary Fig. 2a), and the cellular localization of Integrator subunits was not affected if BRAT1 was deleted (Supplementary Fig. 2b). Notably, even if we employed more input material in our BRAT1 immunopreciptations, we recovered only very low levels of other Integrator complex subunits such as INTS1 and INTS4 (Supplementary Fig. 2c). BRAT1 was detected in INTS4 immunoprecipitates, however to much lesser extent than INTS11 (Supplementary Fig. 2d). These results suggest that BRAT1 tightly interacts with INTS9 and INTS11 subunits, but may be a part of the whole Integrator complex only weakly and/or transiently. Interestingly, we noted that levels of the catalytically active INTS11 subunit were significantly decreased in *BRAT1*^*−/−*^ cells when compared to wild-type cells, implying that BRAT1 may stabilize INTS11 and thus promote Integrator functions (Fig. 1e and Supplementary Fig. 2e).

### Loss of BRAT1 impairs 3′ end processing of UsnRNAs and Cajal body integrity

**Fig. 2:**
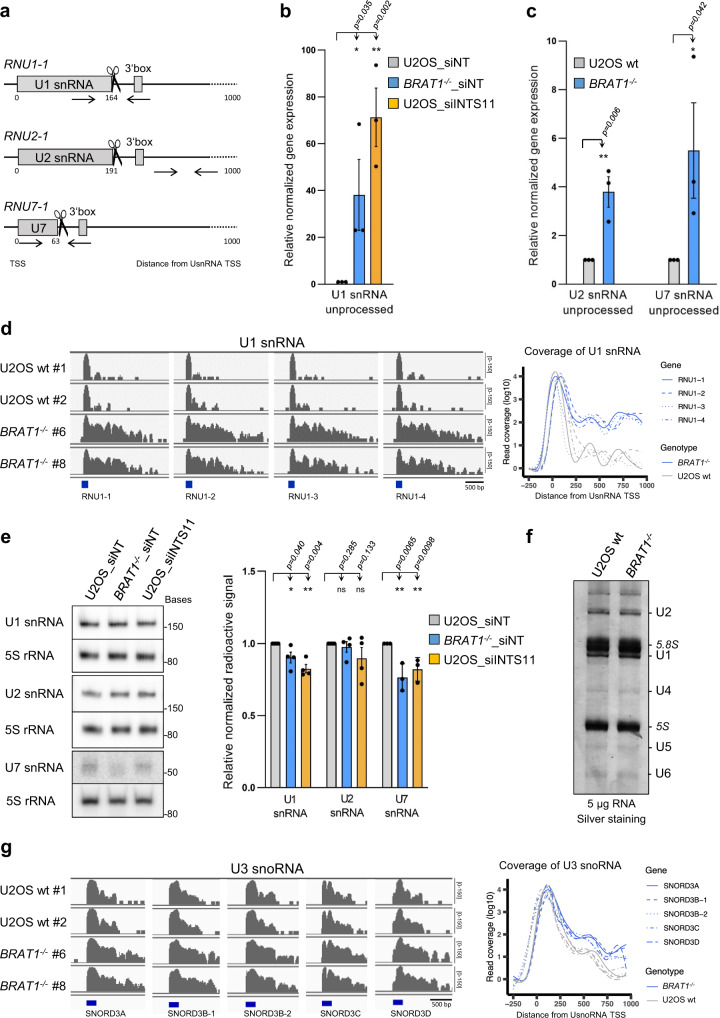
Loss of BRAT1 impairs 3′ end processing of capped UsnRNAs and snoRNAs. **a** Schematic representation of binding sites for primers used for RT-qPCR (*arrows*), canonical Integrator cleavage sites (*scissors*), and a downstream 3´box. **b** RT-qPCR analysis of unprocessed *RNU1-1* transcripts in U2OS and *BRAT1*^*−/−*^ (clone #8) cells transfected with control non-targeting siRNA (siNT) or siINTS11, as indicated. Data are represented as the mean ± SD (*n* = 3). Statistical significance was determined by a one-sided paired Student’s *t*-test (*p < 0.05, **p < 0.01). **c** RT-qPCR analysis of unprocessed *RNU2-1* or *RNU7-1* transcripts in wild-type (U2OS wt) and *BRAT1*^*−/−*^ (clone #8) cells. Data are represented as the mean ± SD (*n* = 3). Statistical significance was determined by a one-sided paired Student’s *t*-test (*p < 0.05, **p < 0.01). **d** RNA-seq data for genes coding U1 snRNA transcripts in wild-type U2OS (wt #1 and wt #2) and *BRAT1*^*−/−*^ (clone #6 and #8) cells. Read coverage is shown on the *left* and metaplot analysis of library-normalized averaged samples on the *right*. TSS transcription start site. See also Supplementary Fig. 3a. **e** Total RNA was isolated from U2OS and *BRAT1*^*−/−*^ (clone #8) cells transfected with control non-targeting siRNA (siNT) or siINTS11, as indicated, resolved on urea-PAGE, Northern blotted and probed for levels of U1, U2, U7 snRNA, and 5S rRNA. Representative blots and quantification are shown. Data are represented as the mean ± SD (*n* = 4 for U1 and U2; *n* = 3 for U7). Statistical significance was determined by a one-sided paired Student’s *t*-test (*p < 0.05, **p < 0.01, ns not significant). **f** Total RNA was isolated from wild-type (U2OS wt) and *BRAT1*^*−/−*^ (clone #8) cells, resolved on urea-PAGE, and silver-stained. The experiment was performed three times with similar results. Uncropped and unprocessed scans are provided in the Source Data file. **g** RNA-seq data for genes coding U3 snoRNA transcripts presented similarly as in **d**.

To investigate the impact of BRAT1 deletion on 3’ end processing of non-polyadenylated RNAPII-dependent uridine-rich small nuclear RNAs (UsnRNAs), we performed quantitative reverse-transcription PCR (RT-qPCR) using sets of primers specific to unprocessed UsnRNAs encoded by human *RNU1-1, RNU2-1* and *RNU7-1* genes (Fig. 2a). As expected, based on previous reports^33,34^, siRNA depletion of INTS11 in U2OS cells induced a pronounced accumulation of unprocessed U1 snRNAs, when compared to control siRNA (Fig. 2b). More importantly, unprocessed U1, U2 and U7 snRNAs were also significantly increased in *BRAT1*^*−/−*^ cells (Fig. 2b, c). Moreover, we detected the accumulation of longer uncleaved forms of U1 snRNA, and a variety of other snRNAs, in *BRAT1*^*−/−*^ cells by RNA-sequencing (RNA-seq) (Fig. 2d and Supplementary Fig. 3a). Importantly, however, there was no change in the U6 snRNA primary transcript that is transcribed by RNA polymerase III (RNAPIII) and is not a substrate of Integrator (Supplementary Fig. 3a). Perhaps surprisingly, despite the differences in UsnRNA processing, there were only slight changes in the levels of mature UsnRNAs in *BRAT1*^*-/-*^ cells, as was the case for INTS11-depleted cells, and which were significant only for U1 and U7 (Fig. 2e, f). Finally, we also detected increased expression of long unprocessed U3 small nucleolar RNAs (U3 snoRNAs) in *BRAT1*^*−/−*^ cells (Fig. 2g). Unlike most snoRNAs, U3 snoRNA genes are transcribed as independent units and have been shown to be processed by the Integrator complex^35^.

**Fig. 3:**
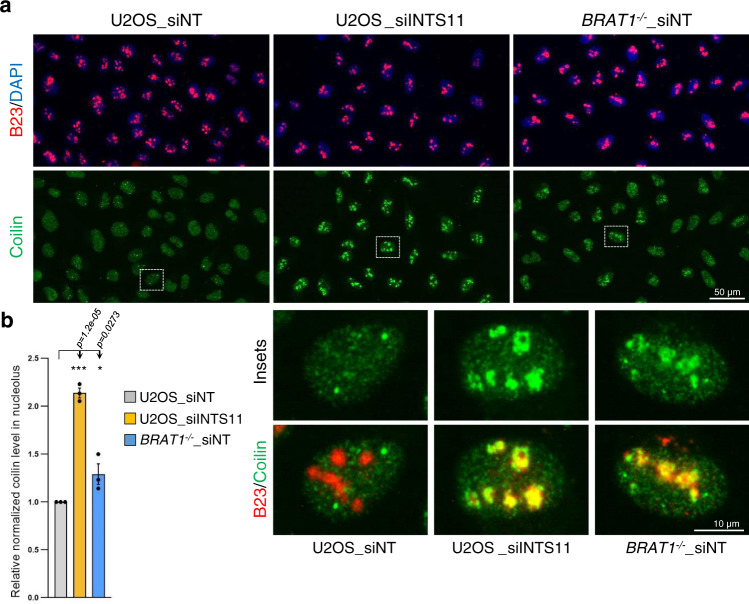
BRAT1 deletion disrupts the structural integrity of Cajal bodies. **a** Immunofluorescence staining of subnuclear structures in U2OS and *BRAT1*^*−/−*^ (clone #8) cells transfected with control non-targeting siRNA (siNT) or siINTS11, as indicated. Cajal bodies were visualized by immunostaining of coilin (*green*), nucleoli by B23 (*red*), and nuclei by DAPI (*blue*). **b** Quantification of normalized coilin fluorescence inside the nucleolus using ScanR software; representative pictures are shown in **a**. Data are represented as the mean ± SD (*n* = 3). Statistical significance was determined by a one-sided paired Student’s *t*-test (*p < 0.05, ***p < 0.001). See also Supplementary Fig. 3b, c.

Since proper processing of UsnRNAs is essential for the integrity of Cajal bodies (CBs), subnuclear structures involved in UsnRNAs biogenesis, we examined whether the defects in UsnRNA processing present in *BRAT1*^*−/−*^ cells might disrupts these structures^36^. We therefore subjected U2OS cells to immunostaining for coilin, an established marker of CBs. CBs detected with coilin were clearly visible in cells transfected with control siRNA (Fig. 3a). However, we observed disruption of CBs and subsequent relocalization of coilin into nucleoli upon INTS11 depletion, consistent with previous reports (Fig. 3a, b)^23,37^. Importantly, disruption of CBs and a concomitant relocalization of coilin into nucleoli was also evident in *BRAT1*^*−/−*^ cells, although to a lesser extent, consistent with a requirement for BRAT1 in maintaining CBs integrity (Fig. 3a, b and Supplementary Fig. 3b, c).

### BRAT1 deletion reduces the efficiency of replication-dependent histone pre-mRNA processing

**Fig. 4:**
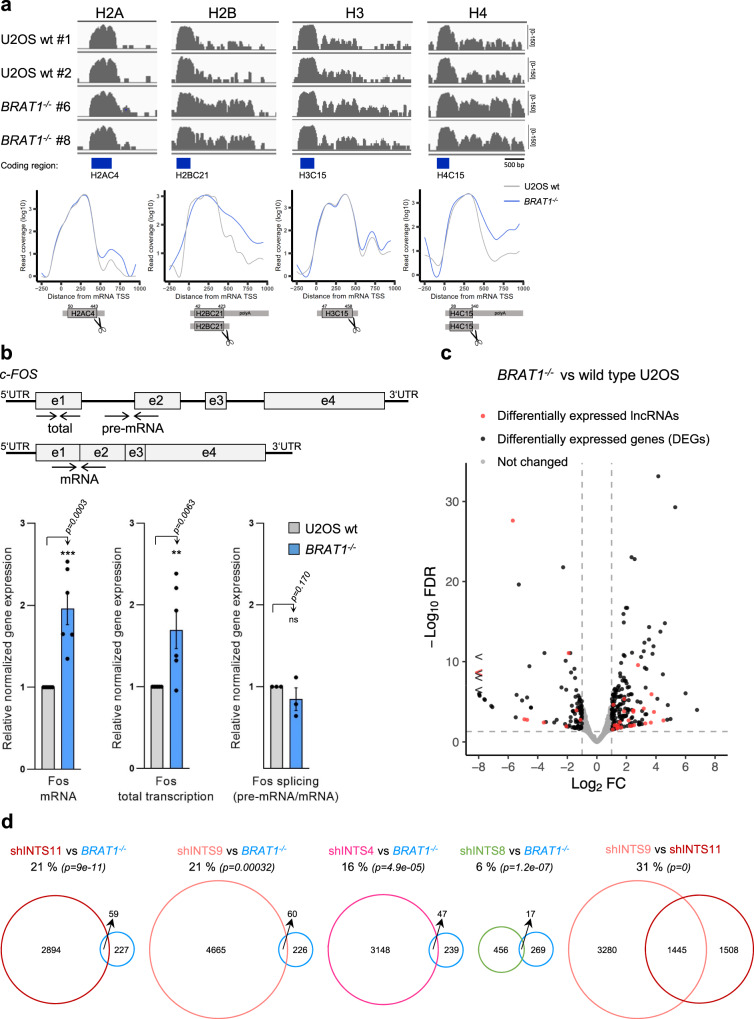
BRAT1 deletion alters biogenesis of replication-dependent histone mRNAs and transcriptional regulation of protein-coding genes. **a** RNA-seq data for genes coding histones H2A, H2B, H3, or H4 in wild-type U2OS (wt #1 and wt #2) and *BRAT1*^*−/−*^ (clone #6 and #8) cells, as indicated. Read coverage and metaplot analysis of averaged samples and histone coding region, 5’UTR, 3’UTR with a cleavage site (*scissors*) are shown. TSS transcription start site. See also Supplementary Fig. 4. **b** Schematic representation indicating pairs of primers (*arrows*) and RT-qPCR analysis of *c-FOS* mRNA and total RNA (pre-mRNA and mRNA) in wild-type (U2OS wt) and *BRAT1*^*−/−*^ (clone #8) cells. The efficiency of splicing was determined by the ratio between the expression of pre-mRNA and mRNA. Data are represented as the mean ± SD (*n* = 6 for mRNA and total transcription; *n* = 3 for splicing). Statistical significance was determined by a one-sided paired Student’s *t*-test (**p < 0.01, ***p < 0.001, ns not significant). **c** A volcano plot of *BRAT1*^*−/−*^ (clone #8 and #6) vs. wild-type U2OS (wt #1 and wt #2) transcription profiles showing differentially expressed genes (DEGs) and differentially expressed lncRNAs. Log_2_ ratio for upregulated genes ≥1.0 (FDR < 0.05) and for downregulated genes ≥ −1.0 (FDR < 0.05), *n* = 27,006. **d** Venn diagrams demonstrating the overlap in identified differentially expressed genes and lncRNAs among cells depleted of BRAT1 (*BRAT1*^*−/−*^), INTS11, INTS9, INTS4, or INTS8 (shRNA transfected cells). Statistical significance of the overlap was determined by a hypergeometric test (*p*-values are indicated).

As shown above, BRAT1 depletion resulted in change in levels of U7 snRNA (Fig. 2c, e), a factor critical for efficient replication-dependent histone 3’ end processing^38^. We therefore explored the impact of BRAT1 deficiency on the processing of DNA replication-dependent histone mRNAs, by interrogating our RNA-seq data for the length of histone transcripts in *BRAT1*^*−/−*^ cells. We found increased levels of longer unprocessed mRNAs for all four core canonical histones H2.A, H2.B, H3 and H4 (Fig. 4a). In contrast, the mRNAs of histone variants such as H3.3, H2AX, and CENP-A, which are replication-independent, were unaffected (Supplementary Fig. 4a)^39^. To confirm the impact of BRAT1 on replication-dependent histone mRNA 3’ end formation, we performed RT-qPCR across the region of the histone H4 transcript that is normally cleaved. Indeed, we detected the accumulation of histone H4 mRNAs that extended beyond the normal site of 3’ end processing, consistent with our RNA-seq data (Supplementary Fig. 4b). Since there were no apparent changes in the coverage of the coding regions, we conclude that BRAT1 depletion impairs 3’ end cleavage without altering the total amount of properly processed replication-dependent histone mRNAs. Interestingly, aberrantly 3’ end processed and/or polyadenylated replication-dependent histone mRNAs have been detected in small amounts in cells after downregulation of a number of factors, including those that regulate transcription elongation^40–43^. Thus, the loss of BRAT1 might impair the histone pre-mRNA processing through the decreased levels of U7 snRNA and/or by finetuning the Integrator activity necessary for the efficient transition from the initial stage of transcription to processive transcription.

### The impact of BRAT1 deletion on transcriptional regulation of protein-coding genes

To investigate potential defects in the regulation of protein-coding genes upon the BRAT1 deletion, we first measured the expression of *c-FOS*, an immediate-early gene that is negatively regulated by Integrator^30,44^. RT-qPCR measurements in *BRAT1*^*−/−*^ cells indicated that the basal expression of this gene was upregulated ~ 2-fold, compared to wild-type cells (Fig. 4b). The impact of BRAT1 on *c-FOS* expression was not due to a defect in splicing, because this was not affected (Fig. 4b). Rather, these data are consistent with the importance of BRAT1 for the efficient premature termination of *c-FOS* transcription, as has been reported for Integrator. Interestingly, of the 286 DEGs identified in *BRAT1*^*−/−*^ U2OS cells by RNA-sequencing, 237 were protein-coding genes and 33 lncRNAs (Fig. 4c and Supplementary Data 2). 156 DEGs (66%) were upregulated by a log_2_ ratio of at least 1.0 (FDR < 0.05) and 81 DEGS (34%) were downregulated by a log_2_ ratio of at least −1.0 (FDR < 0.05). We therefore compared our RNA-seq data with those reported previously for INTS11-, INTS9-, INTS4- and INTS8-depleted human cells^29,45^. Notably, 16–21% of DEGs detected in BRAT1-deleted cells were also differentially expressed in cells depleted of INTS4, INTS9, or INTS11, which are the known components of the Integrator core catalytic cleavage module (Fig. 4d). Interestingly, this level of overlap is not very different from that detected (31%) between INTS9-depleted and INTS11-depleted cells. In contrast, only 6% of DEGs in BRAT1-deficient cells overlapped with those in INTS8-depleted cells, suggesting once again that BRAT1 is more closely related to the core Integrator cleavage subunits INTS9 and INTS11. Together, these data indicate that depletion of BRAT1, INTS9 and INTS11 leads to deregulation of a similar set of genes and that BRAT1 binding to heterodimer INTS9/INTS11 promotes the function of Integrator at these loci.

### Altered Integrator function in BRAT1-mutated patient cells

**Fig. 5:**
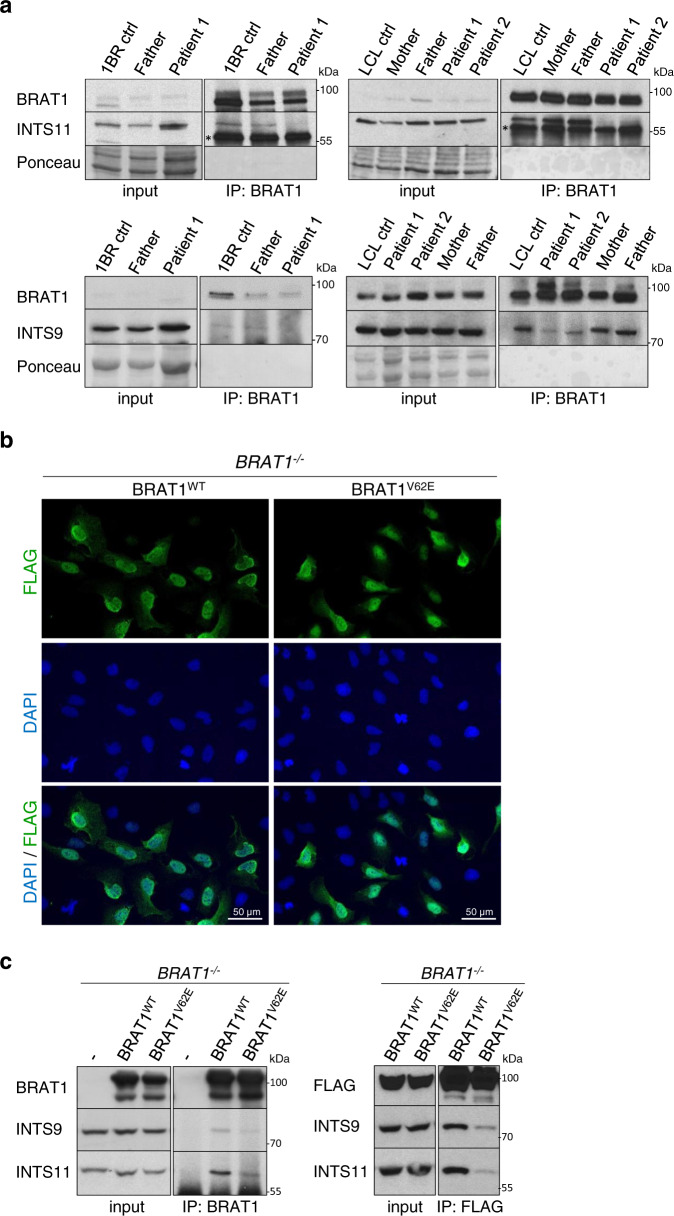
The V62E mutation in BRAT1-mutated patient cells impairs BRAT1 interaction with INTS11 and INTS9. **a** Western blot of BRAT1, INTS11 and INTS9 levels in BRAT1 immunoprecipitates from control, parent, BRAT1 patient-derived fibroblasts and lymphoblastoid cell lines (LCLs), as indicated. Note that 2-fold more lysate from patient cells was employed for loading (input) and immunoprecipitation to ensure equivalent levels of INTS11. The *black asterisk* marks a nonspecific band. **b** *BRAT1*^*−/−*^ (clone #8) cells transiently transfected with FLAG-tagged BRAT1^WT^ and BRAT^V62E^ constructs were fixed 24 h post-transfection and stained with antibody against FLAG-tag (*green*) and DAPI (*blue*). **c** Levels of BRAT1, INTS11, INTS9 and FLAG-tagged BRAT1 in BRAT1 (*left*) and FLAG (*right*) immunoprecipitates from *BRAT1*^*−/−*^ (clone #8) cells transiently transfected with FLAG-BRAT1^WT^ or FLAG-BRAT^V62E^, measured by Western blotting. **a**–**c** The experiment was performed three times with similar results. Uncropped and unprocessed scans are provided in the Source Data file.

The role of the Integrator complex in neurodegeneration is largely unexplored, and there is only one patient described to date with mutations in this complex (in INTS13) that manifests with some cerebellar atrophy^46^. We therefore examined Integrator function in BRAT1-mutated cells derived from patients in which cerebellar atrophy and ataxia is the primary pathology^11^. Strikingly, as observed in *BRAT1*^*−/−*^ U2OS cells, BRAT1 patient-derived lymphoblastoid cell lines (LCLs) and fibroblasts both exhibited significantly reduced INTS11 levels (Supplementary Fig. 5a, b). More importantly, the BRAT1 mutation in this disease (p.V62E) greatly reduced or ablated the interaction of BRAT1 with INTS11 and INTS9 in both patient cell types (Fig. 5a). To further confirm our data, we expressed FLAG-tagged BRAT1^WT^ and BRAT^V62E^ in *BRAT1*^*−/−*^ U2OS cells. Similar to endogenous BRAT1, both BRAT1^WT^ and BRAT1^V62E^ proteins localized preferentially to the nucleus (Fig. 5b) and the V62E mutation markedly decreased the interaction with INTS9 and INTS11, as measured by their reduced co-immunoprecipitation with either BRAT1 or FLAG antibody (Fig. 5c).

**Fig. 6:**
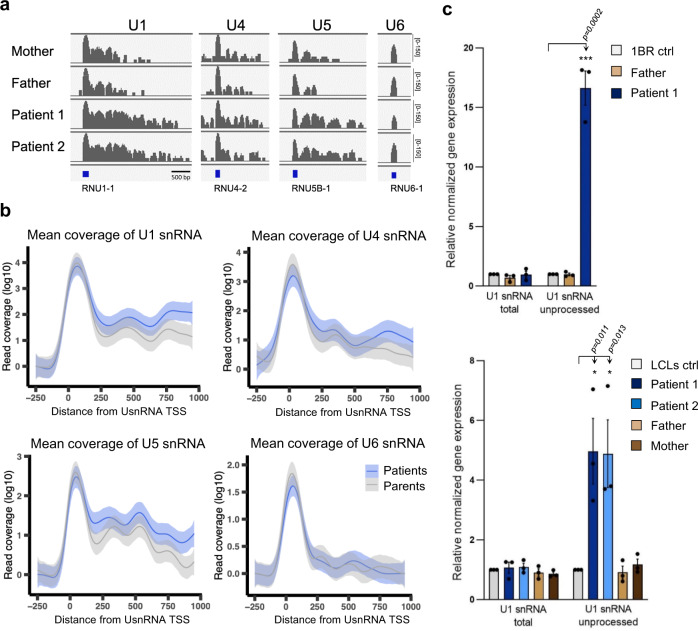
Altered Integrator function in BRAT1-mutated patient cells. **a** RNA-seq coverage plots for genes coding U1, U4, U5, or U6 snRNA in unaffected parents (Mother and Father) and BRAT1 patient-derived (Patient 1 and Patient 2) lymphoblastoid cell lines (LCLs) are shown, as indicated. **b** Metaplot analysis of mean reads coverage of individual U1, U4, U5, or U6 snRNA transcripts from unaffected parents (Mother and Father) and BRAT1 patient-derived (Patient 1 and Patient 2) LCLs. Data are presented as the smoothed mean coverage of the selected genes (*n* = 4) averaged across samples (*n* = 2) in the respective group with 95% confidence interval of the mean shown (*ribbon*). TSS transcription start site. **c** RT-qPCR analysis of total and unprocessed *RNU1-1* transcripts in control, parent and BRAT1 patient-derived fibroblasts (*top*) and LCLs (*bottom*). Data are represented as the mean ± SD (*n* = 3). Statistical significance was determined by a one-sided paired Student’s *t*-test (*p < 0.05, ***p < 0.001).

To characterize the effect of the *BRAT1* mutation on Integrator activity in the patient cells, we performed RNA-seq of LCLs derived from two affected siblings (Patient 1/Patient 2, both harboring the homozygous BRAT1 mutation, V62E) and two unaffected parental controls (Mother/Father, both heterozygous for V62E). Similar to *BRAT1*^*−/−*^ cells, RNA-seq identified differentially expressed protein-coding genes and lncRNAs in BRAT1 patient LCLs, with 62% of DEGs upregulated and 38% downregulated (Supplementary Fig. 5c and Supplementary Data 3). More importantly, and once again similar to *BRAT1*^*−/−*^ cells, whilst we did not detect significant changes in the level of mature UsnRNAs in BRAT1-mutated patient cells (Supplementary Fig. 5d, e), we did detect increased levels of longer unprocessed forms of U1, U4, and U5 snRNA transcripts (Fig. 6a, b). In the case of U1 snRNA, we verified this defect using RT-qPCR in both patient-derived fibroblasts and patient-derived LCLs (Fig. 6c).

In summary, we show here that the BRAT1 patient mutation disrupts the BRAT1 interaction with INTS9/INTS11 heterodimer of the Integrator complex, resulting in destablization of the INTS11 endonuclease subunit and defects in Integrator functions. Thus, based on the pathology of the BRAT1 patients in this study, we further link the improper processing of Integrator RNA substrates to cerebellar atrophy and ataxia.

## Discussion

Germline mutations in *BRAT1* (BRCA1-associated ATM activator 1) result in various neurological pathologies including neurodevelopmental delay and neurodegeneration^4–10^. Recently, we identified a homozygous missense c.185T > A (p.V62E) variant in *BRAT1* that greatly reduced the level of BRAT1 protein in patient-derived cell lines^11^. These patients present clinically with cerebellar atrophy, ataxia, ocular motor apraxia and mild cognitive impairment; a phenotype much milder in comparison to another known BRAT1 related disorders. Although the normal cellular function/s of BRAT1 are unclear, the phenotypes of the associated disease are reminiscent of those present in patients with defective DNA damage responses, and BRAT1 has been reported to interact with the DNA damage response proteins BRCA1 (breast cancer 1) protein and ATM (ataxia-telangiectasia mutated)^1,47^. The latter is particularly intriguing, since ataxia-telangiectasia is also associated with neurodegenerative pathology, including cerebellar ataxia. However, our previous work failed to detect a defect in ATM kinase activation in p.V62E BRAT1 patient cells during the DNA damage response^11^, suggesting that BRAT1 has another functional role, distinct from ATM activation, which if affected similarly leads to neurological disease.

Excitingly, we have now identified BRAT1 as a binding partner of a catalytic RNA endonuclease heterodimer INTS9/INTS11 of Integrator, a multi-subunit protein complex that is conserved across metazoans, is functionally associated with RNA polymerase II (RNAPII) and plays a critical role in processing of a wide array of nascent RNA species (e.g., UsnRNAs, eRNAs, lncRNAs and specific pre-mRNAs)^15,25,30,31,48^. Interestingly, the predicted three-dimensional structure of BRAT1 reveals the presence of HEAT (Huntingtin-Elongation factor 3-protein phosphatase 2A-yeast kinase TOR1) tandem repeats that are a signature motif of a variety of scaffolding proteins. Consistent with this idea, BRAT1 deficiency or the p.V62E BRAT1 mutation present in the BRAT1 patient cells employed here impaired the interactions of BRAT1 with INTS9/INTS11 cleavage heterodimer and significantly reduced the level of INTS11 protein. Collectively, our data best fit a model in which BRAT1 interacts with and stabilizes INTS11, thereby maintaining the structural and functional integrity of the Integrator core cleavage heterodimer. BRAT1 binds other Integrator subunits more weakly or only transiently and might thus be a critical factor, which helps to assemble and efficiently activate the mature Integrator complex at the specific nascent RNA species.

Our data suggest that BRAT1 might promote INTS11 incorporation into the complex and thus control the Integrator functions, such as efficient processing of U1, U2, U4, U5, U7, U11, U12 snRNAs, and U3 snoRNAs. Consistent with this, and similar to core Integrator subunits^23,37^, the presence of BRAT1 in the cells is also required for the integrity of Cajal bodies; prominent nuclear structures that regulate essential cellular processes^49^. This likely reflects the accumulation of misprocessed UsnRNAs and improper UsnRNAs maturation in BRAT1-defective cells, leading to disassembly of Cajal bodies and subsequent progressive relocalization of coilin into nucleoli. Moreover, defects in the metabolism of UsnRNAs have been previously linked to brain disorders^50–52^ and more recently, patients with mutations in the Integrator subunits INTS1 and INTS8 have been identified, which were associated with defects in brain development^13,14^. Altogether, these data indicate that, although BRAT1 and other Integrator complex subunits are ubiquitously expressed, the brain is disproportionally affected by their disruption, suggesting the specific importance for efficient BRAT1-Integrator-dependent RNA processing in long-lived postmitotic neurons. Moreover, complete loss of the BRAT1 protein or most Integrator complex subunits is probably incompatible with human life. Indeed, loss of any Integrator complex component tested to date is lethal in diverse animal models at early developmental stages^33,53–58^.

Importantly, we detected deregulated expression of protein-coding genes in BRAT1-deficent cells, suggesting that BRAT1 is also relevant for other Integrator functions such as cleavage of RNAs associated with paused RNA polymerase II (RNAPII). BRAT1 appears to stabilize the INTS11 endonuclease subunit, thus promoting Integrator-dependent cleavage of specific RNAPII-associated transcripts at the promoter-proximal pausing sites, a process critical for the release of stalled RNAPII facilitating further cycles of transcription elongation^29^. The impact of BRAT1 deficiency on differential expression of protein-coding genes was most probably not primarily due to a defect in splicing because there were no major changes in the levels of mature UsnRNAs in BRAT1-defective cells, however, this might differ in neurons and needs further study. Given the broad roles that BRAT1 and Integrator likely play during RNAPII-mediated transcription of noncoding RNAs and some pre-mRNAs, we hypothesize that the disease pathologies induced by BRAT1 and other Integrator mutations are the combined outcome of an accumulation of unprocessed UsnRNAs and deregulated transcription and/or splicing efficiency, particularly in the brain.

In summary, we propose that the BRAT1 protein is an important factor for stability of the INTS11 endonuclease and/or its incorporation into the Integrator complex and supports its activity. In addition, we show that cells derived from patients with inherited neurodegenerative disease in which BRAT1 is mutated are associated with defects in the Integrator that result in accumulation of long unprocessed snRNAs and incorrect expression of specific RNAs and proteins most likely leading to disease pathology.

## Methods

### Cell lines and culture

Patient-derived hTERT-immortalized fibroblasts (*denoted* Patient 1) and lymphoblastoid cell lines (LCLs; *denoted* Patient 1 and Patient 2) generated from the affected siblings harboring a homozygous missense c.185T > A (p.V62E) variant in *BRAT1*, the control cells from the unaffected parents, both heterozygous for c.185T > A (*denoted* Father and Mother) and the unrelated control fibroblasts (*denoted* 1BR ctrl) or LCLs (*denoted* LCLs ctrl) have been described previously^11^. Written informed consent was obtained at the time the skin biopsies were performed to derive cell lines for future studies by the investigators. Human fibroblasts were cultured in Minimum Essential Medium Eagle (MEM; Gibco) supplemented with 15% fetal bovine serum (FBS; Gibco), 2 mM l-glutamine (Gibco), and the antibiotics *penicillin* (100 units/ml) and *streptomycin* (100 μg/ml) (Pen/Strep; Gibco) at 37 °C. LCLs were grown in Roswell Park Memorial Institute 1640 Medium (RPMI 1640; Sigma) supplemented with 10% FBS and the antibiotics Pen/Strep at 37 °C. Human wild-type osteosarcoma cells (U2OS) were cultured in Dulbecco’s Modified Eagle’s Medium-high glucose (DMEM-high glucose; Sigma) supplemented with 10% FBS and the antibiotics Pen/Strep at 37 °C.

### Generation of *BRAT1*^*−/−*^ cell lines

*BRAT1*^*−/−*^ gene edited U2OS cell lines were prepared using Cas9 and guide sequences designed in CRISPR direct (https://crispr.dbcls.jp):

gRNA1_1 TTTCTTGGCTTTATATATCTTGTGGAAAGGACGAAACACCGTGCTGTTCTGGTAGATCCC, gRNA1_2 GACTAGCCTTATTTTAACTTGCTATTTCTAGCTCTAAAACGGGATCTACCAGAACAGCAC,

gRNA2_1 TTTCTTGGCTTTATATATCTTGTGGAAAGGACGAAACACCGCTGCAGGAGCACCCCTGCC, gRNA2_2 GACTAGCCTTATTTTAACTTGCTATTTCTAGCTCTAAAACGGCAGGGGTGCTCCTGCAGC,

gRNA3_1 TTTCTTGGCTTTATATATCTTGTGGAAAGGACGAAACACCGGCCCAGGTTGCTCGGCCGA, gRNA3_2 GACTAGCCTTATTTTAACTTGCTATTTCTAGCTCTAAAACTCGGCCGAGCAACCTGGGCC.

The selected CRISPR guide oligonucleotide pairs were annealed and extended into a 98-mer double-stranded fragment using Phusion polymerase (Sigma) and subcloned into the guide RNA cloning vector (Addgene; 41824) using Gibson Assembly (NEB). For gene editing, human U2OS cells were co-transfected by the appropriate guide oligonucleotide duplex and a Cas9 expression construct (Addgene; 41815) using Lipofectamine LTX (Life Technologies). Twenty-four hours later, the transfected cells were selected in a medium containing 0.5 mg/ml G418 (Roche) for 5 days. The obtained subclones were analyzed for expression of BRAT1 by indirect immunofluorescence and/or Western blotting. Finally, three clones were chosen for confirmation of gene editing by Sanger sequencing (clones #6, #8, and #16). Genomic DNA was isolated from wild-type U2OS cells and selected clones using QIAamp DNA mini kit (Qiagen; 51304) and PCR was performed to amplify regions of interest surrounding the specific BRAT1 guide RNA target loci using following pairs of primer:

BRAT1_gRNA1F-TGCAGTACAGACCTCTGG and

BRAT1_gRNA1R-AGTCTGCCACAGATAATCC,

BRAT1_gRNA3F-TGCCTCAGCCTTCTTAGTAGC and

BRAT1_gRNA3R-AGACATCGCACAGACCAGAC.

Amplicons were cloned into pCR2.1-TOPO plasmid by Topo TA cloning kit (Life Technologies; 45-0641) according to the manufacturer’s instructions prior to DNA sequencing with M13 forward primer (Eurofins).

### Cloning and site-directed mutagenesis

Full-length wild-type human BRAT1 denoted BRAT1^WT^ (UniProtKB: NP_ 689956.2, Q6PJG6) was cloned into the pDONR221 donor vector (Invitrogen; 12536017) via the BP recombination reaction as indicated by the manufacturer’s instructions (Invitrogen; 11789013). By Sanger sequencing verified pDONR221_BRAT1^WT^ donor construct was used for insertion of BRAT1^WT^ into the pMM330 entry vector via the LR recombinant reaction as indicated by the manufacturer (Invitrogen; 11791019). The resulting pMM330_BRAT1^WT^ expression plasmid thus comprises a TEV-cleavable TwinStrep-FLAG-tag linked in-frame to the N-terminus of the BRAT1 sequence. To prepare BRAT1^V62E^ construct, the site-directed mutagenesis was carried out by Q5 Site-Directed Mutagenesis Kit (BioTech; E0552S) according to the manufacturer’s protocol using mutagenic primers (BRAT1_V62E_F: CTGTCCCATGAGCTGAAAGTCCAGG and BRAT1_V62E_R: CAGCTCCACCAGGCAGGG) and the pMM330_BRAT1^WT^ plasmid as a template. Desired pMM330_BRAT1^WT^ and pMM330_BRAT1^V62E^ constructs were verified by Sanger sequencing.

### Plasmid DNA and siRNA transfection

*BRAT1*^*−/−*^ (clone #8) U2OS cells were transfected by pMM330_BRAT1^WT^ or pMM330_BRAT1^V62E^ expression plasmids using a jetPRIME transfection reagent (Polyplus-transfection; 101000046) according to the manufacturer’s instructions. Transfected cells were fixed or collected for the experiments 24 h later. For siRNA-mediated depletion, U2OS cells were transfected with non-targeting siRNA (siNT; Dharmacon; D-001810-10 or Life Technologies; 4390843), SMART pool siRNA against INTS11 (siINTS11; Dharmacon; SO-2821252G) or siRNA against INTS9 (siINTS9; Life Technologies; 4392420) using Lipofectamine RNAiMAX (Life Technologies) as indicated by the manufacturer. Experiments were carried out 48 h post-transfection.

### Antibodies

Primary antibodies used in this study were as follows: anti-BRAT1 (IF 1:500, WB 1:50,000; Abcam, ab181855), anti-INTS11 (WB 1:1000; Novus Biologicals, NB100-60638), anti-INTS11 (IF 1:500; Novus Biologicals, NBP3-03680), anti-INTS9 (WB 1:1000; Cell Signalling, 13945), anti-INTS4 (WB 1:1000; Abcam, ab75253), anti-INTS3 (WB 1:1000; Bethyl, A302-050A), anti-INTS1 (WB 1:1000; Bethyl, A300-361A), anti-β-actin (WB 1:5000; Protein Tech, 66009), anti-α-tubulin (WB 1:8000; Abcam, ab6160), anti-Lamin B (WB 1:500; Santa Cruz, sc-6216), anti-Coilin (IF 1:250, WB 1:1000; Santa Cruz, sc-32860), anti-B23 (IF 1:250; Santa Cruz, sc-271737) and anti-FLAG (IF 1:250, WB 1:500; Sigma, F1804). Secondary antibodies employed for western blotting were HRP-conjugated goat anti-rabbit (1:10,000; Bio-Rad, 170-6515), goat anti-mouse (1:10,000; Bio-Rad, 170-6516), rabbit anti-rat (1:10,000; Abcam, ab6734), for western blotting after immunoprecipitation HRP-conjugated light chain specific mouse anti-rabbit (1:10,000; Jackson Immunoresearch, 211-032-171) and for indirect immunofluorescence were goat anti-rabbit Alexa 488 (1:10,000; Invitrogen, A-11008) and donkey anti-mouse Alexa 647 (1:10,000; Invitrogen, A-31571).

### SDS-PAGE and western blotting

Cells were collected and lysed in SDS sample buffer (2% SDS, 10% glycerol, 50 mM Tris-HCl, pH 6.8), denaturated for 10 min at 95 °C, and sonicated for 30 s using a Bioruptor® Pico (Diagenode). Protein concentrations were determined using the BCA assay (Pierce; 23227). DTT and bromophenol blue were added to samples, which were subjected to SDS-PAGE, proteins transferred onto nitrocellulose membrane and detected by the relevant primary antibody combined with horseradish peroxidase-conjugated secondary antibody. Induced peroxidase activity was detected using ECL reagent (GE Healthcare) and Amersham Hyperfilm ECL (GE Healthcare). The Amersham Hyperfilm ECL with detected protein signal was scanned and the digital image was analyzed using Image Studio Lite version 5.2 software (LI-COR Biosciences). The intensity of signal was normalized against the loading control (β-actin, α-tubulin, coilin or Lamin B).

### Immunoprecipitation

Cells were washed twice with ice-cold PBS (Gibco) and lysed in 800 µl of ice-cold EBC buffer (50 mM Tris-HCl pH 7.5, 1 mM EDTA, 150 mM NaCl, 0.5% IGEPAL CA-630) supplemented with inhibitors of both proteases and phosphatases (Roche). Cell extracts were sonicated for 30 s using Q120 Sonicator (Qsonica) with 20% amplitude on ice followed by centrifugation at 20,000 × *g* for 10 min at 4 °C. Supernatants were incubated with 1 μg of relevant antibody overnight at 4 °C. Next day, immunoprecipitated complexes were immobilized on protein A/G UltraLink Resin (Life Technologies; 53132) for 2 h at 4 °C and washed by EBC buffer. Similarly, FLAG-tagged immunoprecipitates were incubated with anti-FLAG M2 affinity gel resin (Sigma; SLCH0130) for 2 h at 4 °C and washed by EBC buffer. Bound proteins were subjected to mass spectrometry or eluted with 2x SDS sample buffer and analyzed by SDS-PAGE and western blotting.

### Mass spectrometry and data analysis

Immunoprecipitates from wild-type and *BRAT1*^*−/−*^ (clone #8) U2OS cells were resuspended in 100 mM triethylammonium bicarbonate containing 2% sodium deoxycholate. Cysteins were reduced with 10 mM final concentration of tris(2‐carboxyethyl)phosphine and blocked with 20 mM final concentration of S-methylmethanethiosulfonate (60 °C for 30 min). Samples were cleaved on beads with 1 μg of trypsin overnight at 37 °C. After digestion samples were centrifuged and supernatants were collected and acidified with trifluoroacetic acid to 1% final concentration. Sodium deoxycholate was removed by extraction to ethylacetate^59^. Peptides were desalted using in-house made stage tips packed with C18 disks (Empore)^60^. Nano Reversed phase column (EASY-Spray column, 50 cm × 75 µm ID, PepMap C18, 2 μm particles, 100 Å pore size) was used for LC/MS analysis. Mobile phase buffer A was composed of water and 0.1% formic acid. Mobile phase B was composed of acetonitrile and 0.1% formic acid. Samples were loaded onto the trap column (Acclaim PepMap300, C18, 5 μm, 300 Å Wide Pore, 300 μm x 5 mm, 5 Cartridges) for 4 min at 15 μl/min. Loading buffer was composed of water, 2% acetonitrile and 0.1% trifluoroacetic acid. Peptides were eluted with Mobile phase B gradient from 4% to 35% B in 60 min. Eluting peptide cations were converted to gas-phase ions by electrospray ionization and analyzed on a Thermo Orbitrap Fusion (Q-OT- qIT, Thermo). Survey scans of peptide precursors from 350 to 1400 *m*/*z* were performed at 120 K resolution (at 200 *m*/*z*) with a 5 × 10^5^ ion count target. Tandem MS was performed by isolation at 1.5 Th with the quadrupole, HCD fragmentation with normalized collision energy of 30, and rapid scan MS analysis in the ion trap. The MS/MS ion count target was set to 10^4^ and the max injection time was 35 ms. Only those precursors with charge state 2–6 were sampled for MS/MS. The dynamic exclusion duration was set to 45 s with a 10 ppm tolerance around the selected precursor and its isotopes. Monoisotopic precursor selection was turned on. The instrument was run in top speed mode with 2 s cycles^61^. All data were analyzed and quantified with the MaxQuant software (version 1.6.2.1)^62^. The false-discovery rate (FDR) was set to 1% for both proteins and peptides and we specified a minimum peptide length of seven amino acids. The Andromeda search engine was used for the MS/MS spectra search against the Human database (downloaded from uniprot.org in July 2019, containing 20,444 entries). Enzyme specificity was set as C-terminal to Arg and Lys, also allowing cleavage at proline bonds and a maximum of two missed cleavages. Dithiomethylation of cysteine was selected as fixed modification and N-terminal protein acetylation and methionine oxidation as variable modifications. The “match between runs” feature of MaxQuant was used to transfer identifications to other LC-MS/MS runs based on their masses and retention time (maximum deviation 0.7 min) and this was also used in quantification experiments. Quantifications were performed with the label-free algorithms described recently. Data analysis was performed using Perseus 1.6.1.3 software^63^.

### Cell fractionation

Human cells at 80% confluence were trypsinized and collected by centrifugation at 500 × *g* for 5 min at 4 °C. Pelleted cells were washed twice with ice-cold PBS, followed by resuspension in 5× packed cell volume of ice-cold hypotonic Buffer A (10 mM HEPES-KOH, pH 7.9, 10 mM KCl, 1.5 mM MgCl_2_, 0.5 mM DTT, and 0.5 mM PMSF) supplemented with protease and phosphatase inhibitors (Sigma) and incubation on ice for 5 min. Cells were centrifuged at 500 × *g* for 5 min at 4 °C, resuspended in 2× packed cell volume of supplemented Buffer A. Cells in hypotonic buffer were compressed by Dounce homogenizer 20 times using a tight-fitting pestle in 4 °C. Nuclei were collected by centrifugation at 500 × *g* for 5 min at 4 °C and supernatant was used as a cytoplasmatic fraction. Collected nuclei were lysed in SDS sample buffer (2% SDS, 10% glycerol, 50 mM Tris-HCl, pH 6.8) or ice-cold EBC buffer (50 mM Tris-HCl pH 7.5, 1 mM EDTA, 150 mM NaCl, 0.5% IGEPAL CA-630) and used for western blotting or immunoprecipitation experiments, respectively.

### Immunofluorescence and microscopy

Cells cultured on glass coverslips were fixed in 4% formaldehyde for 10 min and subsequently permeabilized in ice-cold methanol/acetone solution (1:1) for another 10 min. After blocking with 10% FBS for 30 min, fixed cells were incubated with primary antibodies for 60 min, washed in PBS, and then incubated another 60 min with the appropriate fluorescently labeled secondary antibodies. Finally, after washing in PBS, nuclei were stained with DAPI and coverslips were mounted using anti-fading mounting reagent Vectashield (Vector Laboratories). High-resolution microscopy of fixed samples was carried out on Leica DM6000 fluorescence microscope, equipped with dry objectives (Plan-Apochromat 40×/0.75 and 20×/0.70). Automated wide-field microscopy was performed on an Olympus ScanR high-content screening station equipped with a motorized stage and 40x/0.95 (UPLSAPO 2 40×) dry objective. Nucleoli and nuclei were identified based on the B23 and DAPI signal, respectively, and nucleolar/nuclear coilin fluorescence intensity was quantified in the region colocalizing with B23/DAPI using ScanR Analysis Software. The relative coilin level in the nucleolus was calculated as the mean coilin fluorescence intensity in the nucleolus (defined by B23) normalized to the mean fluorescence intensity of coilin in the nucleus (defined by DAPI). At least 600 nuclei were analyzed per condition in three or four independent experiments.

### Quantitative reverse-transcription PCR analysis

Total RNA was extracted from cells using the RNeasy mini kit with an additional DNase I digestion (Qiagen; 74104 and 79254) as indicated by the manufacturer. 1 μg of total RNA was reverse transcribed with RevertAid Reverse Transcriptase (Life Technologies) using random hexamer primers. Quantitative reverse-transcription PCR (RT-qPCR) was performed on a LightCycler 480 (Roche) using SYBR Green PCR Master Mix (Life Technologies) and following target primer pairs:

RNU1_total_F-CAGGGGAGATACCATGATCACGAAG and

RNU1_total_R-GGTCAGCACATCCGGAGTGCAATGG (U1 snRNA);

RNU1_F-GAAACTCGACTGCATAATTTGTGGTAG and

RNU1_R-CTTGGCGTACAGTCTGTTTTTGAAACTC (U1 snRNA unprocessed);

RNU2_F-AACATAGGTACACGTGTGCCACGG and

RNU2_R-ACAAATAGCCAACGCATGCGGGGC (U2 snRNA unprocessed);

RNU7-1_F-CAGTGTTACAGCTCTTTTAG and

RNU7-1_R-ATCATTGGCAACAAACATC (U7 snRNA unprocessed);

HIST1H4B_F-CATCTCAATGGCTTTACTCG and

HIST1H4B_R-ATAGCTCACTTATCTCGGAG (Histone H4 unprocessed);

c-FOS_pre_F-GGCTTTCCCCTTCTGTTTTG and

c-FOS_pre_R-TGGTCGAGATGGCAGTGAC (c-FOS pre-mRNA);

c-FOS_m_F-ACTACCACTCACCCGCAGAC and

c-FOS_m_R-TGGTCGAGATGGCAGTGAC (c-FOS mRNA);

c-FOS_total_F-TACCCAGCTCTGCTCCACAG and

c-FOS_total_R-AGGATGACGCCTCGTAGT (c-FOS exon1);

RPLP2_F-TCTTGGACAGCGTGGGTATCGA and

RPLP2_R-CAGCAGGTACACTGGCAAGCTT (Large Ribosomal Subunit Protein P2);

ACTB_F-CACCATTGGCAATGAGCGGTTC and ACTB_R-AGGTCTTTGCGGATGTCCACGT (Actin Beta).

The expression data were normalized to the data for reference genes ACTB and/or RPLP2. The relative expression was calculated by Pffafl method:

$${{{{{\bf{RQ}}}}}}=\frac{{{{{{{\bf{2}}}}}}}^{\Delta {{{{{\rm{Ct}}}}}}({{{{{{\mathrm{target}}}}}}})}}{{{{{{{\bf{2}}}}}}}^{\Delta {{{{{\rm{Ct}}}}}}({{{{{{\mathrm{reference}}}}}}})}}$$

### Northern blotting

Total RNA was extracted from cells using TRIzol reagent (Life technologies) according to the manufacturer’s protocol and dissolved in urea sample buffer (20 mM Tris-HCl pH 8.0, 8 M urea, 0.2% xylene blue). The extracted total RNA was separated by denaturing 7.5 M urea polyacrylamide gel electrophoresis and transferred by capillarity to a positively charged nylon membrane (GE Healthcare; Amersham HybondTM-N + , RPN303B) overnight at room temperature. Millennium™ RNA Markers (Thermo Fisher Scientific; AM7150) were labeled with [γ-³²P]ATP (Hartmann Analytic; SCP-301) by T4 polynucleotide kinase (Thermo Fisher Scientific; EK0031) up to a final concentration 10^6^ cpm/ml and used in parallel with RNA samples. The blotted RNA was crosslinked to the membrane in a UV Stratalinker 1800 using the short-wave UV light (254 nm, 120 mJ) for 1 min. The crosslinked membrane was pre-hybridized in the Church buffer (1% (w/v) BSA, 1 mM EDTA, 0.5 M Na_2_HPO_4_ * 12 H_2_O, 58.4 mM H_3_PO_4_, 7% SDS) for 2 h at 55 ˚C (U7 snRNA) or 65 ˚C (U1 snRNA, U2 snRNA, 5S rRNA), hybridized with the [γ-³²P]ATP-labeled U1 snRNA, U2 snRNA, U7 snRNA and 5S rRNA probes diluted in the Church buffer up to the final concentration 10^6^ cpm/ml overnight at 55 ˚C (U7 snRNA) or 65˚C (U1 snRNA, U2 snRNA, 5S rRNA), and then washed.

U1 snRNA-CGCAGGGGTCAGCACATCCGGAGTGC,

U2 snRNA-AAATCCATTTAATATATTGTCCTCGG,

U7 snRNA-CTAAAAGAGCTGTAACACTG and

5S rRNA-TCTCCCATCCAAGTACTAACCAGGCCCGACC.

For detection of hybridized target probes, the membrane was exposed to a phosphor screen, which was then scanned by Amersham Typhoon™ biomolecular imager (GE Healthcare). The digital image was analyzed using Image Studio Lite version 5.2 software (LI-COR Biosciences).

### Silver staining of RNA

Total RNA was extracted as above and separated on a 7.5 M urea gel. After fixation with 40% methanol containing 10% acetic acid for 1 h, incubation with 3.4 mM K_2_Cr_2_O_7_ and 3.2 mM HNO_3_ for 10 min and washing, the gel was stained with 12 mM AgNO_3_ for 30 min and developed with 280 mM Na_2_CO_3_ containing 0.02% formaldehyde. The reaction was stopped with 1–5% acetic acid and the stained gel was scanned and analyzed using Image Studio Lite version 5.2 software (LI-COR Biosciences).

### RNA sequencing and data analysis

For the RNA-sequencing experiment, total RNA was isolated from 1 × 10^6^ cells by miRNeasy Micro Kit (Qiagen; 217084) with an additional DNase I treatment, according to the manufacturer’s instructions. The quantity and quality of isolated RNA was measured using NanoDrop ND-2000 (NanoDrop Technologies) and analyzed by Agilent 2100 Bioanalyser (Agilent Technologies). RNA integrity number, which is regarded as criteria for high-quality total RNA, ranged between 9.9 and 10. For each sample, 3 ng of total RNA was used as input material for a library preparation by following the SMARTer Stranded Total RNA-Seq Kit v2—Pico Input Mammalian user manual (Takara Bio USA, Inc; 634412). Libraries were sequenced on the Illumina NextSeq® 500 instrument using 76 bp single-end configuration. Read quality was assessed by FastQC (http://www.bioinformatics.babraham.ac.uk/projects/fastqc). For subsequent read processing, a bioinformatic pipeline nf-core/rnaseq version 1.4.2 was used (nf-core/rnaseq: nf-core/rnaseq version 1.4.2; Zenodo; 10.5281/zenodo.3503887). Individual steps included removing sequencing adaptors and low-quality reads with Trim Galore! (http://www.bioinformatics.babraham.ac.uk/projects/trim_galore/), mapping to reference genome GRCm38 (Ensembl annotation version 98) with HISAT2 and quantifying expression on gene level with featureCounts^64–66^. Per gene mapped counts served as input for differential expression analysis using DESeq2 R Bioconductor package^67^. Prior to the analysis, genes not expressed in at least two samples were discarded. We supplied experimental model assuming sample type (control vs *BRAT1*-defective) as main effect. Resulting per gene expression log2-fold-changes were used for differential expression analysis. Genes exhibiting absolute log2-fold change value of 1 or greater and statistical significance (adjusted *p*-value < 0.05) between compared groups of samples were considered as differentially expressed. Read coverage was visualized using the Integrative Genomics Viewer (IGV).

### Reporting summary

Further information on research design is available in the Nature Research Reporting Summary linked to this article.

## Supplementary information

Supplementary Information

Reporting Summary

Description of Additional Supplementary Files

Supplementary Data File 1

Supplementary Data File 2

Supplementary Data File 3

## Data Availability

The data that support this study are available from the corresponding author upon reasonable request. The MS data are deposited to the ProteomeXchange Consortium via the PRIDE partner repository with the dataset identifier PXD028591. All the raw-sequencing data reported in this paper are available on ArrayExpress with the Accession number E-MTAB-10750. Source data are provided with this paper.

## Source data

Source Data
