# Supplementary material for: BRAT1 links Integrator and defective RNA processing with neurodegeneration
Source: Nat Commun. 2022 Aug 26;13:5026. doi: 10.1038/s41467-022-32763-6 (PMC9418311; doi:10.1038/s41467-022-32763-6)
Supplement: Supplementary file 1 — Supplementary Information [file 41467_2022_32763_MOESM1_ESM.pdf]

Supplementary Figures

BRAT1 Links Integrator and Defective RNA Processing with Neurodegeneration

Zuzana Cihlarova et al.

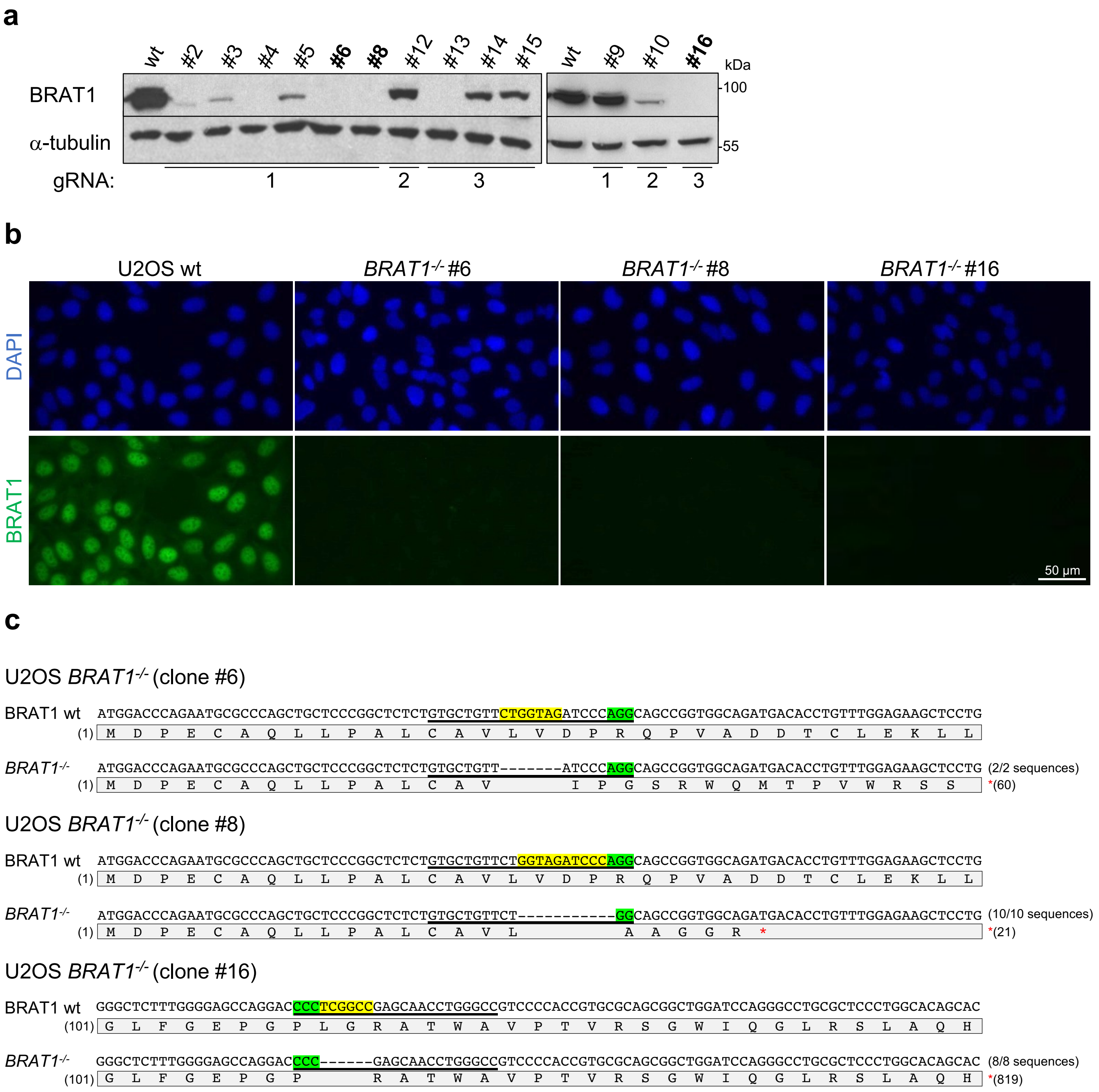

Supplementary Fig. 1

Characterization of *BRAT1* gene-edited cell lines generated by CRISPR/Cas9.

a BRAT1 protein levels in wild-type U2OS (wt) cells and gene-edited clones targeted with different *BRAT1* gRNA, as indicated, were analysed by western blotting. **b** Selected *BRAT1*$^{-/-}$ cell lines (clones #6, #8, and #16) immunostained with BRAT1 antibody. Representative wide-field images are shown. **a, b** The experiment was performed three times with similar results. **c** Confirmation of *BRAT1* gene editing in clones #6, #8, and #16 by PCR amplification of genomic DNA spanning the gRNA target site followed by subcloning and Sanger sequencing. CRISPR/Cas9 guides are underlined in wild type sequences (BRAT1 wt) and the associated PAM is indicated in *green*, deletions in *yellow*, and resulting stop codons are indicated by a *red asterisk*.

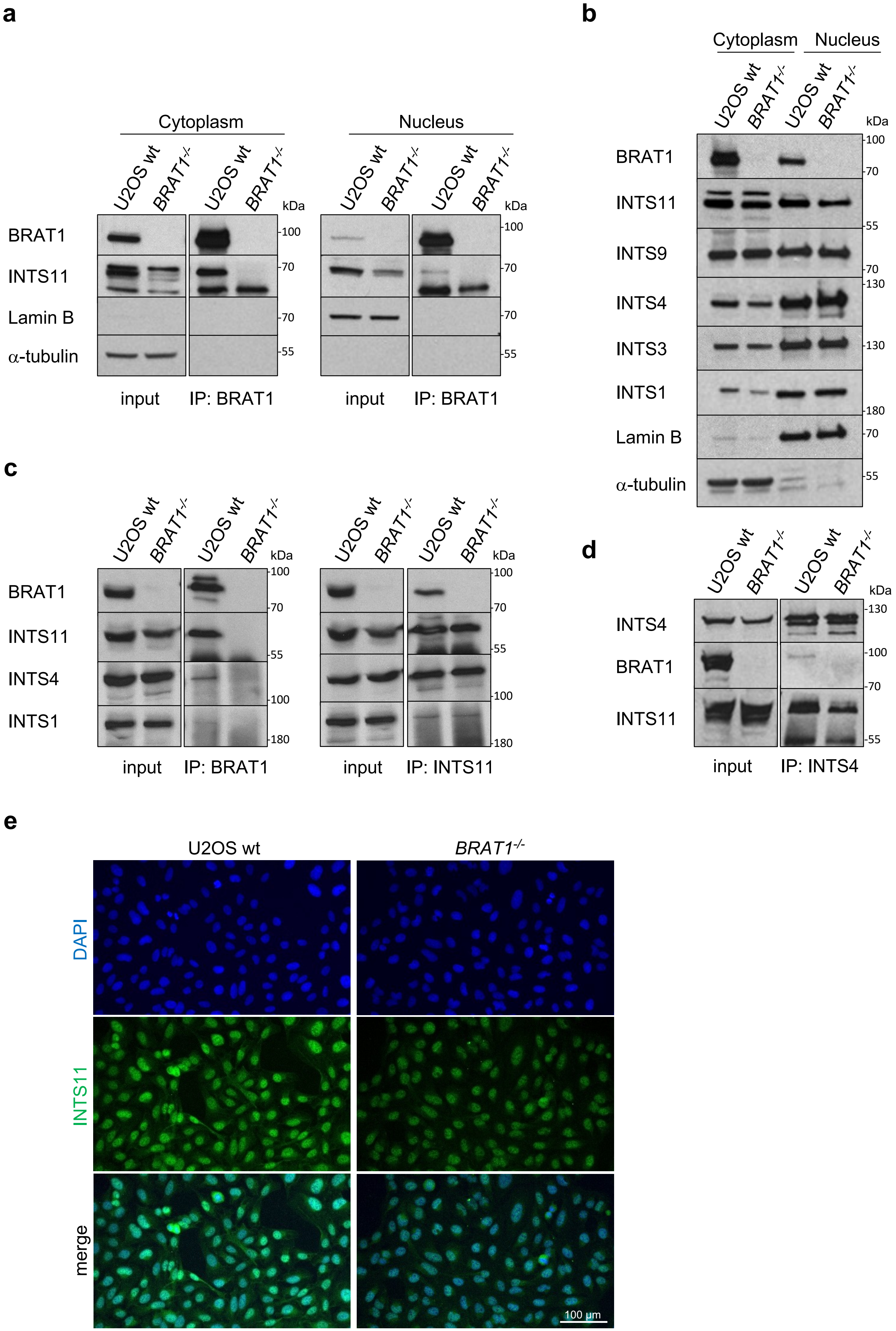
a
Cytoplasm
Nucleus
U2OS wt
BRAT1-/-
U2OS wt
BRAT1-/-
U2OS wt
BRAT1-/-
U2OS wt
BRAT1-/-
kDa
BRAT1
INTS11
Lamin B
α-tubulin
100
70
70
55
input
IP: BRAT1
input
IP: BRAT1
b
Cytoplasm
Nucleus
U2OS wt
BRAT1-/-
U2OS wt
BRAT1-/-
kDa
BRAT1
INTS11
INTS9
INTS4
INTS3
INTS1
Lamin B
α-tubulin
100
70
55
70
130
130
180
70
55
c
U2OS wt
BRAT1-/-
U2OS wt
BRAT1-/-
U2OS wt
BRAT1-/-
U2OS wt
BRAT1-/-
kDa
BRAT1
INTS11
INTS4
INTS1
100
70
55
100
180
input
IP: BRAT1
input
IP: INTS11
d
U2OS wt
BRAT1-/-
U2OS wt
BRAT1-/-
kDa
INTS4
BRAT1
INTS11
130
100
70
55
input
IP: INTS4
e
U2OS wt
BRAT1-/-
DAPI
INTS11
merge
100 µm

Supplementary Fig. 2
BRAT1 interacts with INTS11.
a Levels of BRAT1, INTS11, Lamin B and α-tubulin in BRAT1 immunoprecipitates from cytoplasmic and nuclear fractions of wild type (U2OS wt) and *BRAT1*$^{-/-}$ (clone #8) cells, measured by western blotting. **b** Immunoblot of various Integrator subunits in cytoplasmic and nuclear fractions of wild type (U2OS wt) and *BRAT1*$^{-/-}$ (clone #8) cells, as indicated. Samples derived from the same experiment and blots were processed in parallel. **c** Levels of BRAT1, INTS11, INTS4 and INTS1 in BRAT1 (*left*) and INTS11 (*right*) immunoprecipitates from wild type (U2OS wt) and *BRAT1*$^{-/-}$ (clone #8) cells, measured by western blotting. **d** Levels of INTS4, BRAT1 and INTS11 in INTS4 immunoprecipitates from wild type (U2OS wt) and *BRAT1*$^{-/-}$ (clone #8) cells, measured by Western blotting. **a, b, c, d** The experiment was performed three times with similar results. Uncropped and unprocessed scans are provided in the Source Data file. **e** Immunofluorescence staining of INTS11 (*green*) in wild type (U2OS wt) and *BRAT1*$^{-/-}$ (clone #8) cells. Nuclei were visualized by DAPI (*blue*). The experiment was performed three times with similar results.

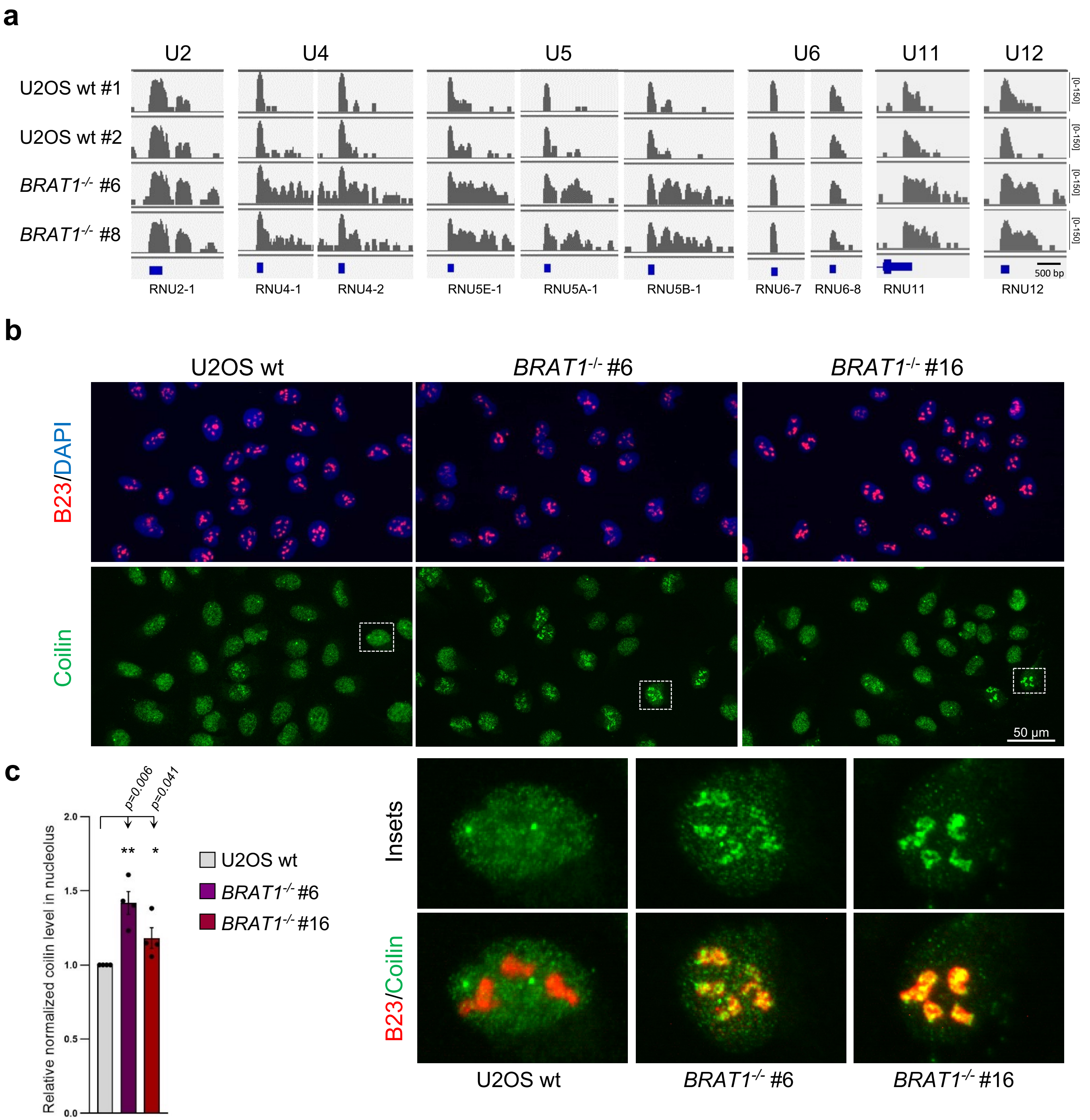

Supplementary Fig. 3

BRAT1 depletion impairs the efficient 3′ end processing of UsnRNAs and the structural integrity of Cajal bodies.

a RNA-seq read coverage for genes coding U2, U4, U5, U6, U11, or U12 snRNA in wild type U2OS (wt #1 and wt #2) and *BRAT1*$^{-/-}$ (clone #6 and #8) cells are shown, as indicated. **b** Immunofluorescence staining of subnuclear structures in wild type (U2OS wt) and *BRAT1*$^{-/-}$ (clone #6 and #16) cells. Cajal bodies were visualized by immunostaining of coilin (*green*), nucleoli by B23 (*red*), and nuclei by DAPI (*blue*). **c** Quantification of normalized fluorescent coilin signal inside the nucleolus using ScanR software; representative pictures are shown in (**b**). Data are represented as the mean ± SD (n = 4). Statistical significance was determined by a one-sided paired Student’s t-test (*p < 0.05, **p < 0.01).

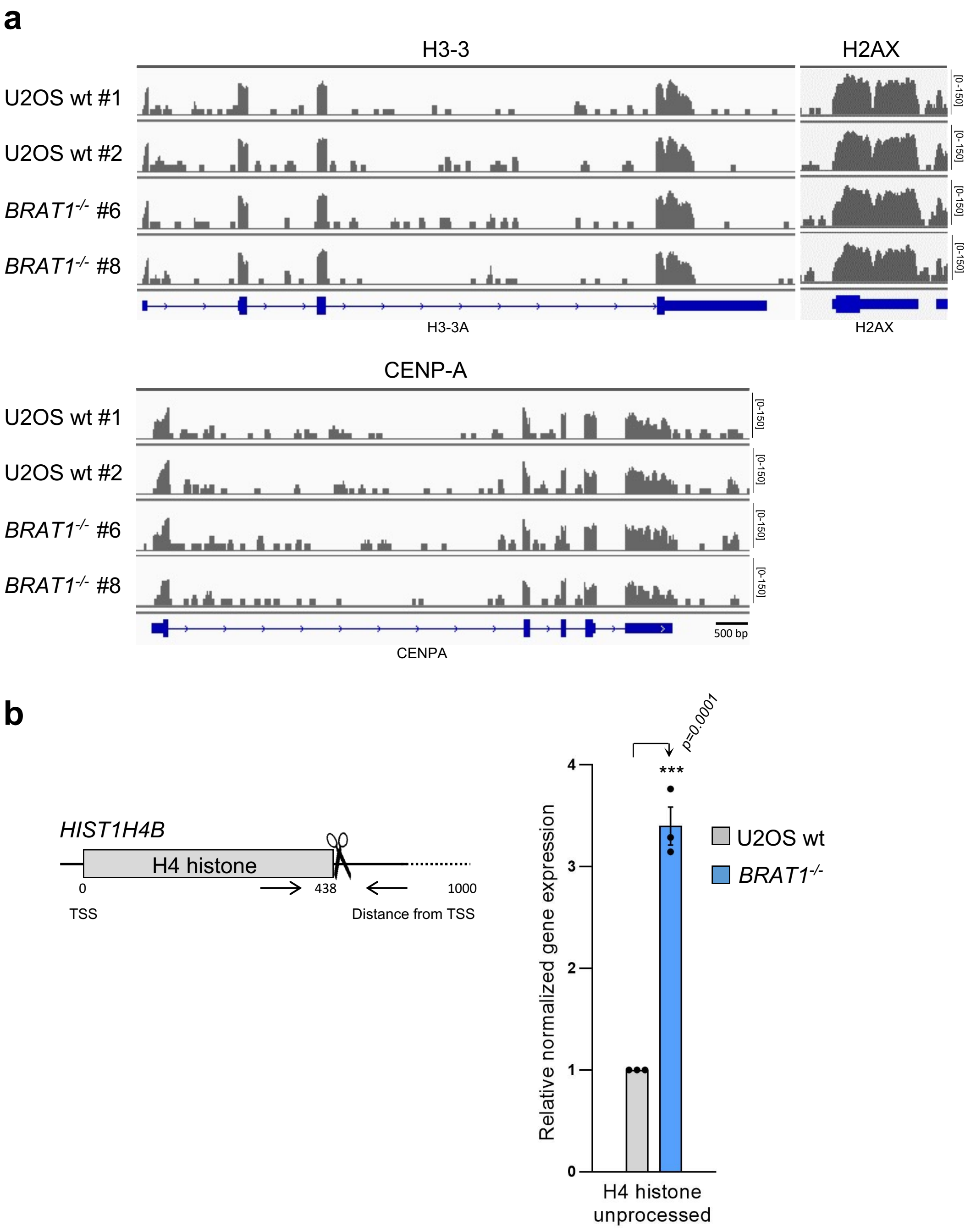

Supplementary Fig. 4

mRNA levels of replication-independent histone variants are unaffected in *BRAT1*$^{-/-}$ cells.

a RNA-seq read coverage for genes coding histones H3-3, H2AX, or CENP-A in wild type U2OS (wt #1 and wt #2) and *BRAT1*$^{-/-}$ (clone #8 and #6) cells are shown, as indicated. **b** Schematic representation of binding sites for primers used for RT-qPCR (*arrows*), a cleavage site (*scissors*), and RT-qPCR analysis of unprocessed *HIST1H4B* transcripts in wild type (U2OS wt) and *BRAT1*$^{-/-}$ (clone #8) cells. Data are represented as the mean ± SD (n = 3). Statistical significance was determined by a one-sided paired Student's t-test (***$p < 0.001$).

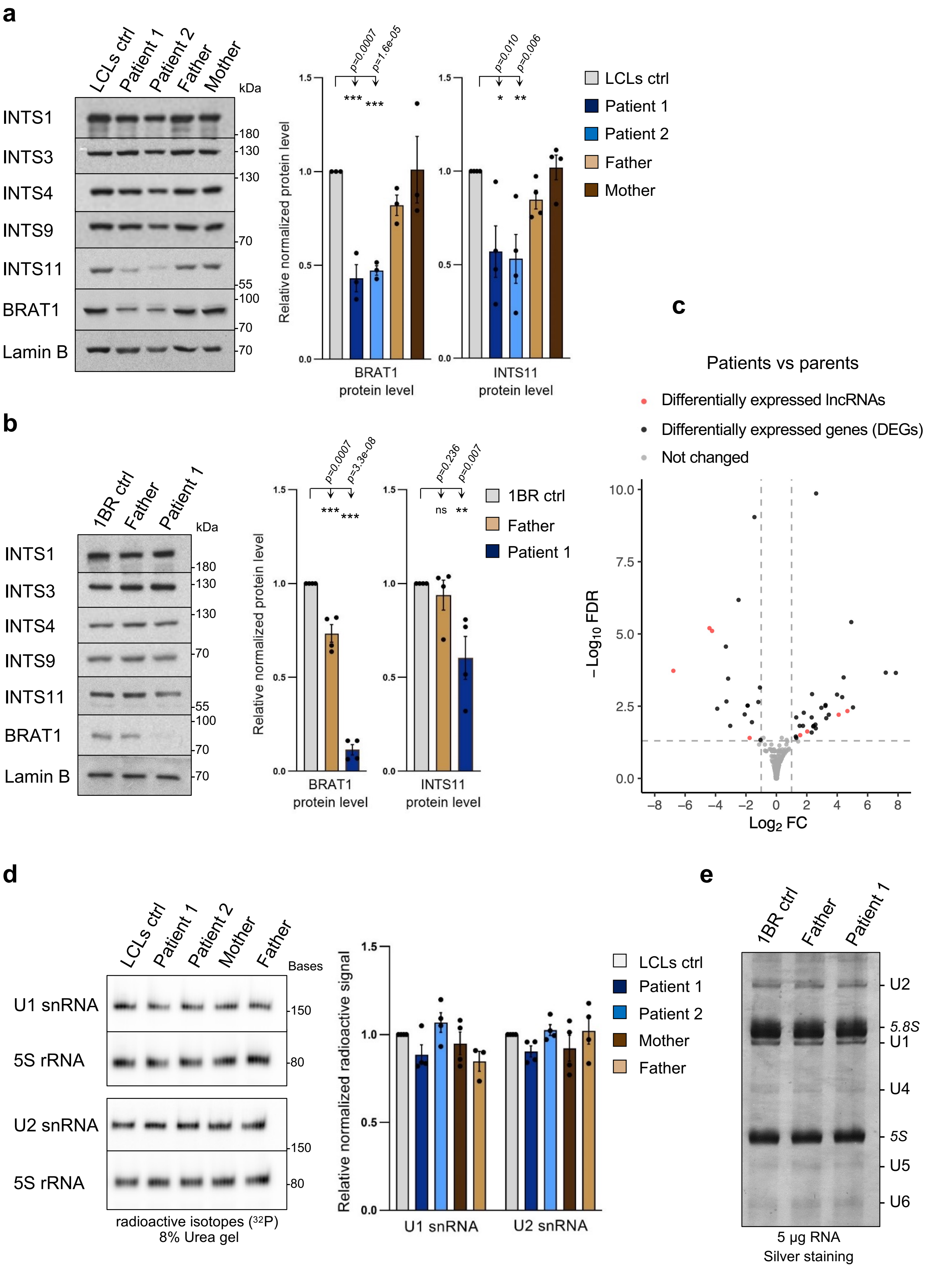
a
LCLs ctrl
Patient 1
Patient 2
Father
Mother
kDa
INTS1
INTS3
INTS4
INTS9
INTS11
BRAT1
Lamin B
180
130
130
70
55
100
70
70
Relative normalized protein level
p=0.0007
p=1.6e-05
***
***
p=0.010
p=0.006
*
**
BRAT1 protein level
INTS11 protein level
LCLs ctrl
Patient 1
Patient 2
Father
Mother
b
1BR ctrl
Father
Patient 1
kDa
INTS1
INTS3
INTS4
INTS9
INTS11
BRAT1
Lamin B
180
130
130
70
55
100
70
70
Relative normalized protein level
p=0.0007
p=3.3e-08
***
***
p=0.236
p=0.007
ns
**
BRAT1 protein level
INTS11 protein level
1BR ctrl
Father
Patient 1
c
Patients vs parents
Differentially expressed lncRNAs
Differentially expressed genes (DEGs)
Not changed
−Log10 FDR
Log2 FC
d
LCLs ctrl
Patient 1
Patient 2
Mother
Father
Bases
U1 snRNA
5S rRNA
U2 snRNA
5S rRNA
150
80
150
80
radioactive isotopes (32P)
8% Urea gel
Relative normalized radioactive signal
U1 snRNA
U2 snRNA
LCLs ctrl
Patient 1
Patient 2
Mother
Father
e
1BR ctrl
Father
Patient 1
U2
5.8S
U1
U4
5S
U5
U6
5 µg RNA
Silver staining

Supplementary Fig. 5

Reduced INTS11 levels and deregulated transcription in BRAT1-mutated patient cells.

a, b Immunoblot and quantification of the indicated proteins in control, parent and BRAT1 patient-derived lymphoblastoid cells (LCLs) (**a**) and fibroblasts (**b**). Data are represented as the mean ± SD (n = 3 for BRAT1; n = 4 for INTS11). Statistical significance was determined by a one-sided paired Student's t-test (*p < 0.05, **p < 0.01, ***p < 0.001, ns - not significant). Samples derived from the same experiment and blots were processed in parallel. **c** Volcano plot of BRAT1-patients (Patient 1 and Patient 2) *vs* parents (Mother and Father) LCLs transcription profiles showing differentially expressed genes (DEGs) and differentially expressed lncRNAs. Log_2 ratio for upregulated genes ≥ 1.0 (FDR < 0.05) and for downregulated genes ≥ -1.0 (FDR < 0.05), n = 28722. **d** Total RNA was isolated from control, parent and BRAT1 patient-derived LCLs, as indicated, resolved on urea-PAGE, Northern blotted and probed against U1, U2 snRNA, and 5S rRNA. Representative blots and quantification are shown. Data are represented as mean ± SD (n = 4). **e** Total RNA was isolated from control (1BR), Father and BRAT1 patient-derived fibroblasts, resolved on urea-PAGE, and silver-stained. **a, b, d, e** Uncropped and unprocessed scans are provided in the Source Data file.

Supplementary Data 1. BRAT1 interacting partners identified by mass spectrometry.
Supplementary Data 2. Differentially expressed genes in *BRAT1$^{-/-}$* cells.
Supplementary Data 3. Differentially expressed genes in BRAT1-mutated patient cells*.*
