# Supplementary material for: BRAT1 links Integrator and defective RNA processing with neurodegeneration
Source: Nat Commun. 2022 Aug 26;13:5026. doi: 10.1038/s41467-022-32763-6 (PMC9418311; doi:10.1038/s41467-022-32763-6)
Supplement: Supplementary file 2 — Reporting Summary [file 41467_2022_32763_MOESM2_ESM.pdf]

nature research

Corresponding author(s): Hana Hanzlikova

Last updated by author(s): Aug 2, 2022

Reporting Summary

Nature Research wishes to improve the reproducibility of the work that we publish. This form provides structure for consistency and transparency in reporting. For further information on Nature Research policies, see Authors & Referees and the Editorial Policy Checklist .

Statistics

For all statistical analyses, confirm that the following items are present in the figure legend, table legend, main text, or Methods section.

n/a	Confirmed	
[ ]	[x]	The exact sample size (*n*) for each experimental group/condition, given as a discrete number and unit of measurement
[ ]	[x]	A statement on whether measurements were taken from distinct samples or whether the same sample was measured repeatedly
[ ]	[x]	The statistical test(s) used AND whether they are one- or two-sided<br>*Only common tests should be described solely by name; describe more complex techniques in the Methods section.*
[x]	[ ]	A description of all covariates tested
[x]	[ ]	A description of any assumptions or corrections, such as tests of normality and adjustment for multiple comparisons
[ ]	[x]	A full description of the statistical parameters including central tendency (e.g. means) or other basic estimates (e.g. regression coefficient) AND variation (e.g. standard deviation) or associated estimates of uncertainty (e.g. confidence intervals)
[ ]	[x]	For null hypothesis testing, the test statistic (e.g. *F*, *t*, *r*) with confidence intervals, effect sizes, degrees of freedom and *P* value noted<br>*Give P values as exact values whenever suitable.*
[x]	[ ]	For Bayesian analysis, information on the choice of priors and Markov chain Monte Carlo settings
[x]	[ ]	For hierarchical and complex designs, identification of the appropriate level for tests and full reporting of outcomes
[x]	[ ]	Estimates of effect sizes (e.g. Cohen's *d*, Pearson's *r*), indicating how they were calculated

Our web collection on statistics for biologists contains articles on many of the points above.

Software and code

Policy information about availability of computer code

Data collection	No software was used for data collection
Data analysis	ScanR Analysis Software (Version 3.2.0), Prism Software (Version 9.4.0 GraphPad), Image Studio Lite version 5.2 software (LI-COR Biosciences)

For manuscripts utilizing custom algorithms or software that are central to the research but not yet described in published literature, software must be made available to editors/reviewers. We strongly encourage code deposition in a community repository (e.g. GitHub). See the Nature Research guidelines for submitting code & software for further information.

Data

Policy information about availability of data

All manuscripts must include a data availability statement. This statement should provide the following information, where applicable:

- Accession codes, unique identifiers, or web links for publicly available datasets
- A list of figures that have associated raw data
- A description of any restrictions on data availability

All data generated or analyzed during this study are included in this article.
The MS data are deposited to the ProteomeXchange Consortium via the PRIDE partner repository with the dataset identifier PXD028591 and 10.6019/PXD028591.
All the raw sequencing data reported in this paper are available on ArrayExpress with the Accession number E-MTAB-10750 and could be accessed under this link: https://www.ebi.ac.uk/arrayexpress/experiments/E-MTAB-10750.

Field-specific reporting

Please select the one below that is the best fit for your research. If you are not sure, read the appropriate sections before making your selection.

[x] Life sciences [ ] Behavioural & social sciences [ ] Ecological, evolutionary & environmental sciences

For a reference copy of the document with all sections, see nature.com/documents/nr-reporting-summary-flat.pdf

Life sciences study design

All studies must disclose on these points even when the disclosure is negative.

Sample size	No statistical methods were used to determine sample size. The sample size follows common standards employing three or more biological replicates, which is based on extensive laboratory experience and literature in the field. Sample size is reported in the legends for all figures.
Data exclusions	No data was excluded
Replication	All findings were successfully replicated at least 3 times
Randomization	Samples were randomly allocated into experimental groups
Blinding	The investigators were blinded to group allocation during data collection and analysis

Reporting for specific materials, systems and methods

We require information from authors about some types of materials, experimental systems and methods used in many studies. Here, indicate whether each material, system or method listed is relevant to your study. If you are not sure if a list item applies to your research, read the appropriate section before selecting a response.

Materials & experimental systems

n/a	Involved in the study
[ ]	[x] Antibodies
[ ]	[x] Eukaryotic cell lines
[x]	[ ] Palaeontology
[x]	[ ] Animals and other organisms
[x]	[ ] Human research participants
[x]	[ ] Clinical data

Methods

n/a	Involved in the study
[x]	[ ] ChIP-seq
[x]	[ ] Flow cytometry
[x]	[ ] MRI-based neuroimaging

Antibodies

Antibodies used	anti-BRAT1 (IF 1:500, WB 1:50000; Abcam, ab181855), anti-INTS11 (WB 1:1000; Novus Biologicals, NB100-60638), anti-INTS11 (IF 1:500; Novus Biologicals, NBP3-03680), anti-INTS9 (WB 1:1000; Cell Signalling, 13945), anti-INTS4 (WB 1:1000; Abcam, ab75253), anti-INTS3 (WB 1:1000; Bethyl, A302-050A), anti-INTS1 (WB 1:1000; Bethyl, A300-361A), anti-a-actin (WB 1:5000; Protein Tech, 66009), anti-g-tubulin (WB 1:8000; Abcam, ab6160), anti-Lamin B (WB 1:500; Santa Cruz, sc-6216), anti-Coilin (IF 1:250, WB 1:1000; Santa Cruz, sc-32860), anti-B23 (IF 1:250; Santa Cruz, sc-271737) and anti-FLAG (IF 1:250, WB 1:500; Sigma, F1804)<br>goat anti-rabbit (1:10000; Bio-Rad, 170-6515), goat anti-mouse (1:10000; Bio-Rad, 170-6516), rabbit anti-rat (1:10000; Abcam, ab6734), mouse anti-rabbit (1:10000; Jackson Immunoresearch, 211-032-171), goat anti-rabbit Alexa 488 (1:10000; Invitrogen, A-11008) and donkey anti-mouse Alexa 647 (1:10000; Invitrogen, A-31571).
Validation	Relevant data for validation of all primary antibodies for western blotting or immunofluorescence are presented on the manafactures websites and/or validated in the current manuscripts using siRNAs (INTS11, INTS9, INTS4) or knock-out cells (BRAT1).

Eukaryotic cell lines

Policy information about cell lines

Cell line source(s)	Patient-derived hTERT-immortalized fibroblasts (denoted Patient 1) and lymphoblastoid cell lines (LCLs; denoted Patient 1 and Patient 2) generated from the affected siblings harbouring a homozygous missense c.185T>A (p.V62E) variant in BRAT1, the control cells from the unaffected parents, both heterozygous for c.185T>A (denoted Father and Mother) and the unrelated control fibroblasts (denoted 1BR ctrl) or LCLs (denoted LCLs ctrl) have been described previously (Mahjoub, A. et al. Neurol Genet 5, e359, doi:10.1212/NXG.0000000000000359, 2019). U2OS cells were obtained from ATCC.

Authentication	These are all authenticated by fingerprinting in our cell culture facility
Mycoplasma contamination	This is confirmed for all cell lines, routinely (every 6 months), in our cell culture facility. All cells were tested negative for mycoplasma contamination.
Commonly misidentified lines (See ICLAC register)	No commonly misidentified cells were used in this study.
