# Supplementary material for: BRAT1 links Integrator and defective RNA processing with neurodegeneration
Source: Nat Commun. 2022 Aug 26;13:5026. doi: 10.1038/s41467-022-32763-6 (PMC9418311; doi:10.1038/s41467-022-32763-6)
Supplement: Supplementary file 3 — Description of Additional Supplementary Files [file 41467_2022_32763_MOESM3_ESM.pdf]

Description of Additional Supplementary Files

Supplementary Data 1. BRAT1 interacting partners identified by mass spectrometry.

Supplementary Data 2. Differentially expressed genes in BRAT1-/- cells.

Supplementary Data 3. Differentially expressed genes in BRAT1-mutated patient cells.
